# Targeting ALDH1A1 to induce Necroptosis in Nasopharyngeal Carcinoma

**DOI:** 10.7150/jca.77914

**Published:** 2022-10-24

**Authors:** Haihua Wang, Yuting Zhan, Shuping Peng, Songqing Fan, Weiyuan Wang

**Affiliations:** 1 Department of Pathology, The Second Xiangya Hospital, Central South University, Changsha, Hunan, 410011, China.; 2 Department of Pathology, The Xiangya Hospital, Central South University, Changsha, Hunan, 410008, China.; 3 Cancer Research Institute, School of Basic Medical Science, Central South University, Changsha, Hunan, 410078, China.

**Keywords:** nasopharyngeal carcinoma, ALDH1A1, NCT-501, necroptosis

## Abstract

ALDH1A1 is one of the highly conserved isoenzymes of the aldehyde dehydrogenase family. It is mainly involved in the metabolism of intracellular aldehydes and forms transcriptional regulators, which are essential for growth and differentiation of normal cells. Overexpression of ALDH1A1 in many malignancies and cancer stem cells (CSCs) is closely associated with poor prognosis and promotes tumor aggressiveness and drug resistance during conventional cancer chemotherapy. In this study, we found that ALDH1A1 had tumor suppressor effects in BRCA, CESC, LIHC, Lung cancer, renal cell carcinoma and PAAD, but tumor-promoting effects in SKCM, GBM, THCA and BLCA. As for the nasopharyngeal carcinoma, ALDH1A1 mainly played a carcinogenic role. We found that although the expression of ALDH1A1 in NPC tissue was lower than that in normal nasopharyngeal mucosal tissue, it was upregulated in patients with higher clinical stages, and correlated with poor patient outcomes. Therefore, we further analyzed the main possible role of ALDH1A1 in NPC by taking GSE12452 dataset. The GSEA enrichment analysis showed that it could inhibit the necroptosis of nasopharyngeal carcinoma cells. Therefore, we used the targeted inhibitor NCT-501 and found that it could inhibit the proliferation and stem cell spheroidization of NPC cells, and induce necroptosis. This study explored the possible role of ALDH1A1 in various tumors and focused on its potential role as a target in NPC. Meanwhile, ALDH1A1 inhibitor preferentially has potential therapeutic value in NPC.

## Introduction

There are a lot of aldehydes in the human body from the external environment and the internal metabolism of biomolecules 1. Aldehydes in sufficiently large amounts can cause cytotoxicity and carcinogenesis 2. Human aldehyde dehydrogenases (ALDHs), composed of 19 nicotinamide adenine dinucleotide phosphate-positive (NAD(P)^+^)-dependent isozymes, metabolize endogenously and exogenously produced aldehydes to their corresponding carboxylic acid derivatives to relieve aldehyde stress 3 The aldehyde dehydrogenase (ALDH) superfamily has distinct tissue or developmental expression 4. The ALDH1/2 family includes five isoenzymes, ALDH1A1, ALDH1A2, ALDH1A3, ALDH2, and ALDH1B1. Among them, ALDH1A1, also known as retinal dehydrogenase 1 (RALDH1), is a highly conserved cytoplasmic homo-tetramer that serves not only as a marker and predictor of prognosis in cancer stem cells, but also plays an important role in the biology of them 5, 6. A key role exhibited by ALDH1A1 is the oxidation of retinal to retinoic acid (RA), forming a transcriptional regulator essential for normal cell growth and differentiation 7. Overexpression of ALDH1A1 in many malignancies and cancer stem cells (CSCs) is strongly associated with poor prognosis, as well as tumor aggressiveness and drug resistance during conventional cancer chemotherapy 8. As one of the broadest and deepest members of ALDH, ALDH1A1 serves as a promising therapeutic target for multiple diseases 3.

Given the broad disease-related functions of ALDH1A1, significant efforts have been made in the academic community to develop potent and selective ALDH1A1 inhibitors. Small molecule ALDH inhibitors are a prudent approach to identify potential cancer cell or CSC-directed therapies and to better understand the physiological and pathophysiological roles of ALDH 9. By inhibiting ALDH1A1 activity, ovarian cancer spheroid formation and cell survival rate are destroyed *in vitro*, as well as the size of xenograft tumor *in vivo* 10. Furthermore, cancer stem cells (CSCs) are increasingly associated with chemoresistance. NCT-501 (an ALDH1A1 inhibitor) inhibits functional properties of cisplatin-resistant cells and induces cell sensitivity in head and neck squamous cell carcinoma 11.

Nasopharyngeal carcinoma (NPC) is one of the most common cancers in southern China. The incidence is as high as 25 per 100,000 people 12. Mortality from NPC remains high due to the development of treatment resistance and distant metastases. This result may be due to the presence of cancer stem cells (CSCs). Previous studies have found that ALDH1 can be used to identify functional markers of cancer stem cells in human nasopharyngeal carcinoma 13. ALDH1A1 is one of the isoenzymes of ALDH. In NPC, its expression is related to the stemness of NPC. It may also be the predominant form of its enzymatic activity and is associated with enhanced cellular invasiveness 14, suggesting that it can also be a potential therapeutic target for NPC.

Necroptosis is a process of programmed cell death which is performed in response to specific stimuli and involves activation of cellular signaling pathways. It has been shown to play a role in malignancy development and confer chemoresistance 15. Therapeutic targets in necroptosis signaling mechanisms and therapeutic strategies capable of effectively inducing necroptotic cell death may help to counteract the development of chemoresistance 16. It has been reported that elevated ALDH inhibits the necroptosis of tumor cells 17. A novel ALDH1A inhibitor induces necroptosis in ovarian cancer cell lines and showed synergy with conventional chemotherapy and was associated with attenuated oxidative phosphorylation 16.

In this study, we found that ALDH1A1 was down-regulated in most tumor cells compared to normal cells, but its role in different tumors was diverse. It was found that although the expression of ALDH1A1 in NPC tissue was lower than that in normal nasopharyngeal mucosal tissue, it was up-regulated in patients with higher clinical stages and correlated with poor prognosis. Therefore, we further divided ALDH1A1 into two groups in the public data set GSE12452, using GSEA enrichment analysis found that it was mainly involved in the inhibition of necroptosis in NPC. Next, we used the ALDH1A1 inhibitor NCT-501, and found that it could inhibit the proliferation and stem cell spheroidization of NPC cells, and induce necroptosis. This study explored the possible role of ALDH1A1 in various tumors, and furtherly studied the potential role of it in NPC. And NCT-501, as a targeted inhibitor of ALDH1A1, had potential therapeutic value in NPC.

## Results

### Overview of ALDH1A1 in pan-cancers

**Figure 1:**
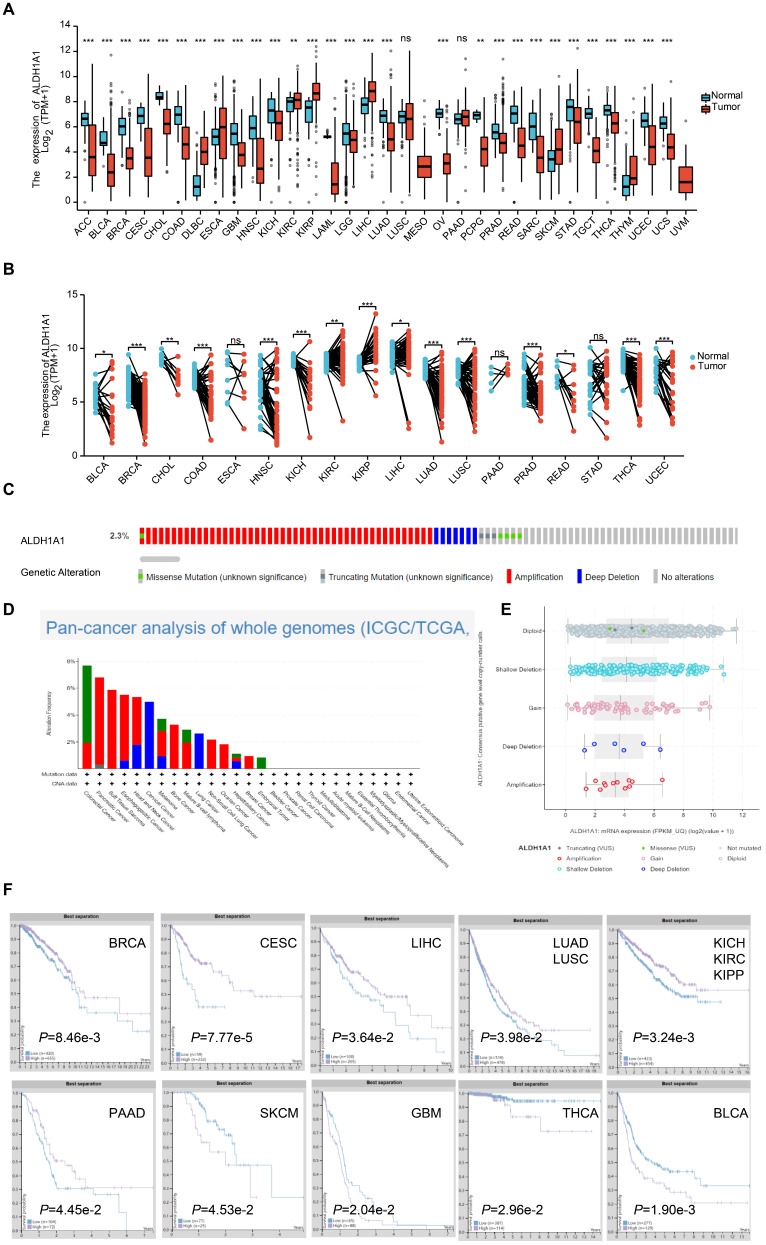
** Overview of ALDH1A1 in pan-cancers. (A)** Transcriptional expression of ALDH1A1 in 33 types of cancer using the TCGA-GTEx database. **(B)** mRNA levels of ALDH1A1 in 18 paired cancers and para-cancerous using TCGA-GTEx database. **(C)** Analysis of the whole genome data of 2683 pan-cancer cases in The Cancer Genome Atlas dataset. **(D)** ALDH1A1 CNV frequency across 27 TCGA cancer types. **(E)** ALDH1A1 mRNA expression in different CNV types. **(F)** Prognostic value of ALDH1A1 protein expression in 10 types of cancer patients.

Studies have found that each ALDH isoform has a specific differential expression pattern, most of which have respective functional roles in cancer prognosis 18. We found the mRNA levels of ALDH1A1 were significantly lower in Adrenocortical carcinoma (ACC), Bladder Urothelial Carcinoma (BLCA), Breast invasive carcinoma (BRCA), Cervical squamous cell carcinoma and endocervical adenocarcinoma (CESC), Cholangiocarcinoma (CHOL), Colon adenocarcinoma (COAD), Glioblastoma multiforme (GBM), Head and Neck squamous cell carcinoma (HNSC), Kidney Chromophobe (KICH), Acute Myeloid Leukemia (LAML), Brain Lower Grade Glioma (LGG), Lung adenocarcinoma (LUAD), Ovarian cancer (OV), Pheochromocytoma and Paraganglioma (PCPG), Prostate adenocarcinoma (PRAD), Rectum adenocarcinoma (READ), Sarcoma (SARC), Stomach adenocarcinoma (STAD), Testicular Germ Cell Tumors (TGCT), Thyroid carcinoma (THCA), Uterine Corpus Endometrial Carcinoma (UCEC) and Uterine Carcinosarcoma (UCS) tumor tissues than that in normal tissues. While it was significantly higher in the Lymphoid Neoplasm Diffuse Large B-cell Lymphoma (DLBC), Esophageal carcinoma (ESCA), Kidney renal clear cell carcinoma (KIRC), Kidney renal papillary cell carcinoma (KIRP), Liver hepatocellular carcinoma (LIHC), Skin Cutaneous Melanoma (SKCM) and Thymoma (THYM) groups of tumor tissues than that in normal tissues. There was no significant difference in the Lung squamous cell carcinoma (LUSC) and Pancreatic adenocarcinoma (PAAD) groups (Figure 1A). ALDH1A1 mRNA levels in 18 tumors and corresponding paired para-tumor tissues were further analyzed. It was found that the mRNA expression level of ALDH1A1 in the BLCA, BRCA, CHOL, COAD, HNSC, KICH, LUAD, LUSC, PRAD, READ, THCA, UCEC was significantly decreased, while the ALDH1A1 levels in the KIRC, KIRP, LIHC groups was up-regulated. There was no significant difference in the expressions of ESCA, PAAD and STAD (Figure 1B). Some of the tumors had differences in the expression between the paired groups and the unpaired groups. Like the ALDH1A1 level of PAAD was up-regulated in the paired group but has no significance in the unpaired group. While, the ALDH1A1 was decreased in the paired LUSC, rather than in the unpaired groups, which may be related to the limited number of samples in the paired group.

Analysis of the whole genome data of 2683 pan-cancer cases in The Cancer Genome Atlas dataset found that amplification, deep deletion, gain, shallow deletion, diploid, truncating mutation, and missense of ALDH1A1 occurred at 2.3% or less, but no complete censoring occurred, and gene amplification did not significantly upregulate the level of the gene.

The relationship between the protein expression level of ALDH1A1 and the prognosis of patients was further analyzed. Patients with low expression of ALDH1A1 in BRCA, CESC, LIHC, LUAD and LUSC, KICH, KIRC, KIPP and PAAD were found to have shorter survival time. However, patients with high expression in SKCM, GBM, THCA and BLCA had poor prognosis.

### The expression of ALDH1A1 is up-regulated in higher clinical stages, and associated with poor prognosis in NPC

**Figure 2:**
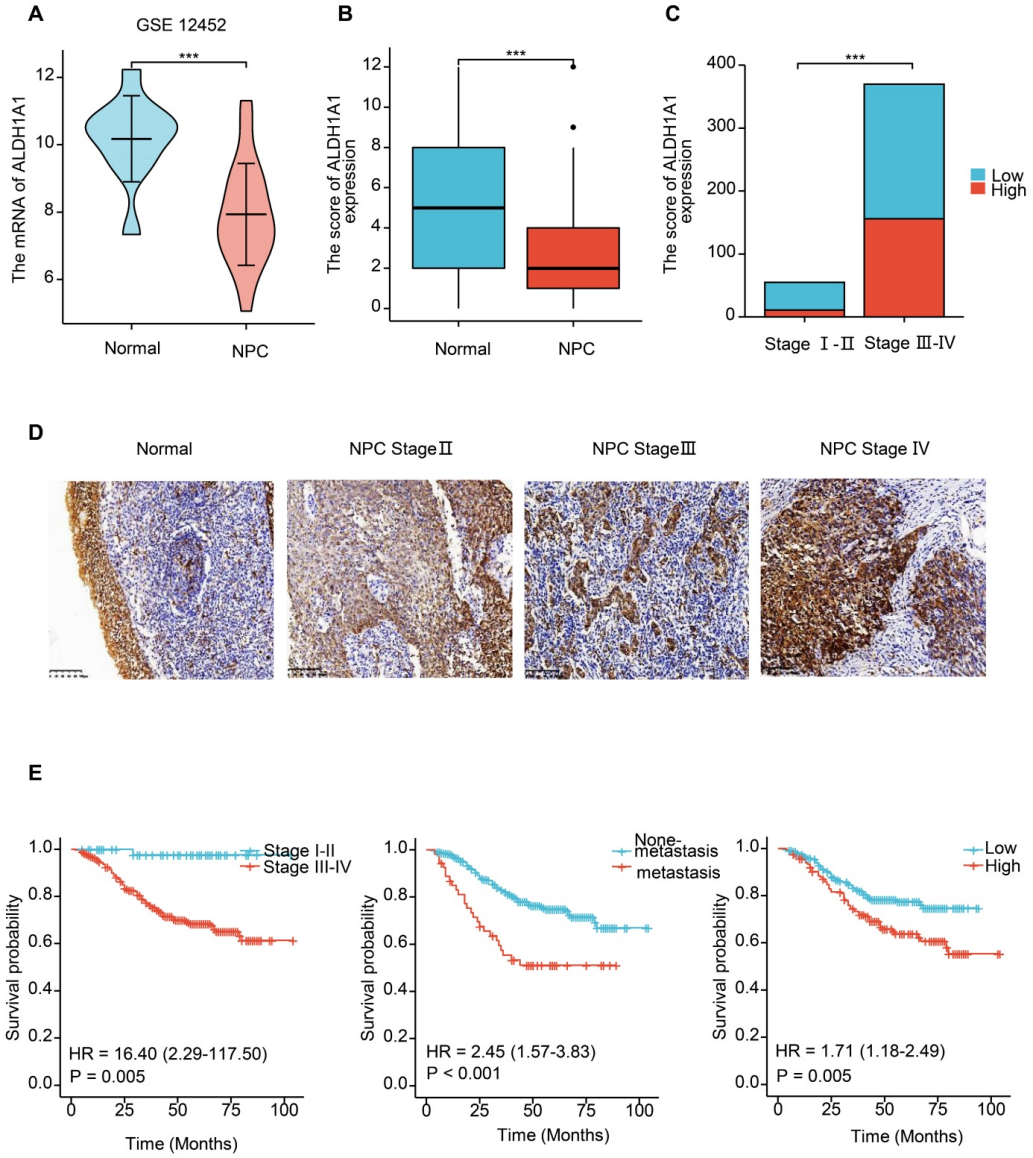
** The expression of ALDH1A1 is up-regulated in higher clinical stages, and associated with poor prognosis in NPC. (A)** Transcriptional expression of ALDH1A1 in NPC tissues and corresponding normal tissues (GEO database, GSE 12452). **(B)** The ALDH1A1 protein expression in NPC tissues and corresponding normal tissues. **(C)** The ALDH1A1 protein expression in clinical stage I-II VS. III-IV. **(D)** The expression of ALDH1A1 in normal nasopharyngeal mucosal tissues and the gradual increasement NPC tissues of different clinical stages. **(E)** The overall survival rates (OS) of NPC patients with different clinical stages, metastasis status and ALDH1A1 expression.

We further analyzed the level of ALDH1A1 in NPC. Analysis of the GEO database found that in the dataset of GSE12452 of NPC, the mRNA level of ALDH1A1 was significantly decreased compared with normal tissues (Figure 2A), which is consistent with the level of ALDH1A1 in most of the other tumors mentioned above. The expression level of ALDH1A1 protein in NPC was further analyzed by immunohistochemistry. It was found that compared with normal nasopharyngeal mucosal epithelium, the expression of ALDH1A1 protein in NPC tissue was consistent with the mRNA level, which was lower in NPC tissues than that in normal nasopharyngeal mucosa (Figure 2B). But it was worth noting that ALDH1A1 expressed at higher levels in higher clinical stages than that in lower one (Figure 2C). The Figure 2D showed it was mainly expressed in the cytoplasm, and the expression of ALDH1A1 in normal nasopharyngeal mucosal tissues and the gradual increasement NPC tissues of different clinical stages.

The relationship between the expression level of ALDH1A1 and the clinical characteristics of patients was further analyzed. It can be seen from the data in Table 1 that the expression of ALDH1A1 had an evidently positive relation with lymph node status, clinical stages and survival status of patients with NPC (*P* = 0.038, *P* = 0.001 and *P* < 0.001, respectively), but there was no relation could be seen in terms of age, gender, tumor stages and metastasis (all *P* > 0.05). Univariate survival analysis (log-rank test) showed that the overall survival rates (OS) was significantly lower for NPC patients with higher clinical stages, metastasis, or elevated ALDH1A1 expression (*P* = 0.005, *P* < 0.001, *P* = 0.005, respectively, Figure 2E). In addition, further analysis using multivariate Cox regression model data showed that higher expression of ALDH1A1 was identified as an independent poorer prognostic factor for patients with NPC (*P* = 0.019), as well as metastasis, and advanced clinical stages (*P* = 0.001 and *P* = 0.011, respectively). No prognostic effects were detected in NPC patients with different age, gender, tumor stages or lymph node status (all *P >* 0.05) (Table 2).

### ALDH1A1 inhibitor can inhibit the ability of NPC stem cells spheroidization and proliferation

**Figure 3:**
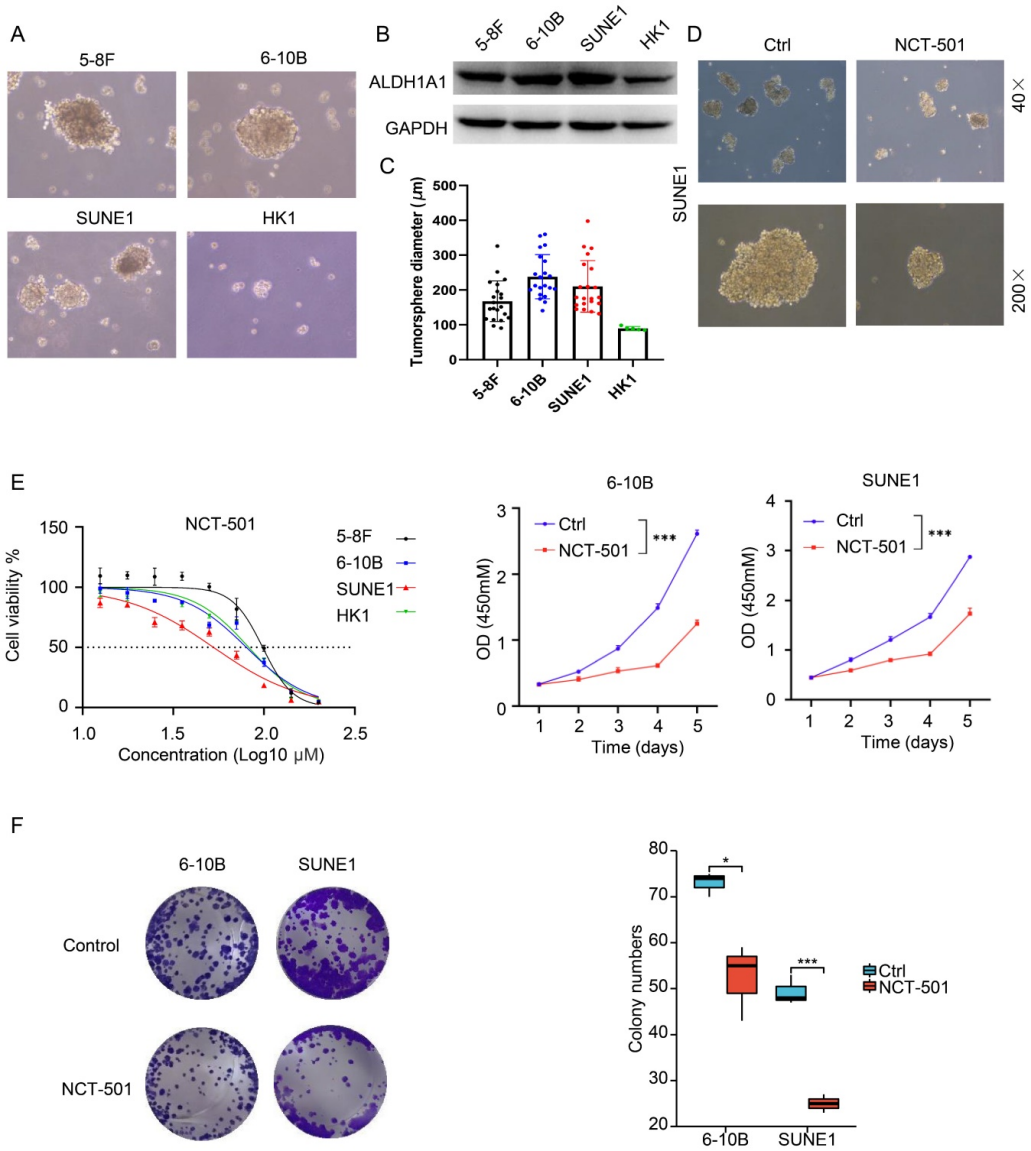
** ALDH1A1 inhibitor can inhibit the ability of NPC of stem cells spheroidization and proliferation. (A)** The stem cells spheroidization of four NPC cells (5-8F, 6-10B, SUNE1 and HK1). **(B)** The expression of ALDH1A1 in four NPC cells. **(C)** The tumor sphere diameter of four NPC cells. **(D)** The stem cells spheroidization of SUNE1 cells treated by NCT-501 and control cells. **(E)** The cell viability of NPC cells treated by NCT-501. And the IC50 of 5-8F, 6-10B, SUNE1 and HK1 were 96.91 µM, 64.15 µM, 27.84 µM and 67.97 µM, respectively. **(F)** The colony formation assay of 6-10B and SUNE1 cells exposed to NCT-501 and control.

ALDH1A1 is widely expressed in various human organs and tissues. In addition to serving as a stemness marker in malignant tumors, ALDH1A1 also exhibits a key role in the oxidation of retinal to retinoic acid (RA), forming a transcriptional regulator critical for normal cell growth and differentiation 19. We detected ALDH1A1 expression and stem cell spheroid of four NPC cell lines, including 5-8F, 6-10B, SUNE1 and HK1. And found that the stem cell spheroid of NPC cells was correlated with the expression level of ALDH1A1. As shown in Figure 3A, among four cell lines, 5-8F, 6-10B and SUNE1 cells had bigger stem cell spheroid diameter within higher ALDH1A1. The expression of ALDH1A1 was lower in HK1 cells, and its stem cell spheroid diameter was also small (Figure 3A-C).

Based on studies of utilizing non-specific ALDH inhibitors and siRNA silencing techniques, inhibition of ALDH1A1 activity might provide new therapeutic options for many diseases, including cancers. NCT-501 is one of the types of quinoline derivatives 20. It can effectively inhibit the activity of ALDH1A1, thereby weakening the drug resistance of drug-resistant cell lines through inhibiting the ability of ovarian cancer cells to form spheroids, showing anti-cancer effect 20. Therefore, we used the ALDH1A1 inhibitor NCT-501 to treat SUNE1 cells, and found that it could inhibit the stem cell spheroid ability of SUNE1 cells (Figure 3D). At the same time, the NCT-501 sensitivity of four NPC cell lines were consistent with the expression level of ALDH1A1 in the cells. When the expression of ALDH1A1 was higher, the cells were more sensitive to NCT-501 (Figure 3E, left). And the IC50 of 5-8F, 6-10B, SUNE1 and HK1 were 96.91 μM, 64.15 μM, 27.84 μM and 67.97 μM, respectively. NCT-501 could also inhibit the proliferation and colony formation (Figure 3E, right and Figure 3F) of 6-10B and SUNE1 cells.

### NCT-501 targeting ALDH1A1 induced necroptosis in NPC

**Figure 4:**
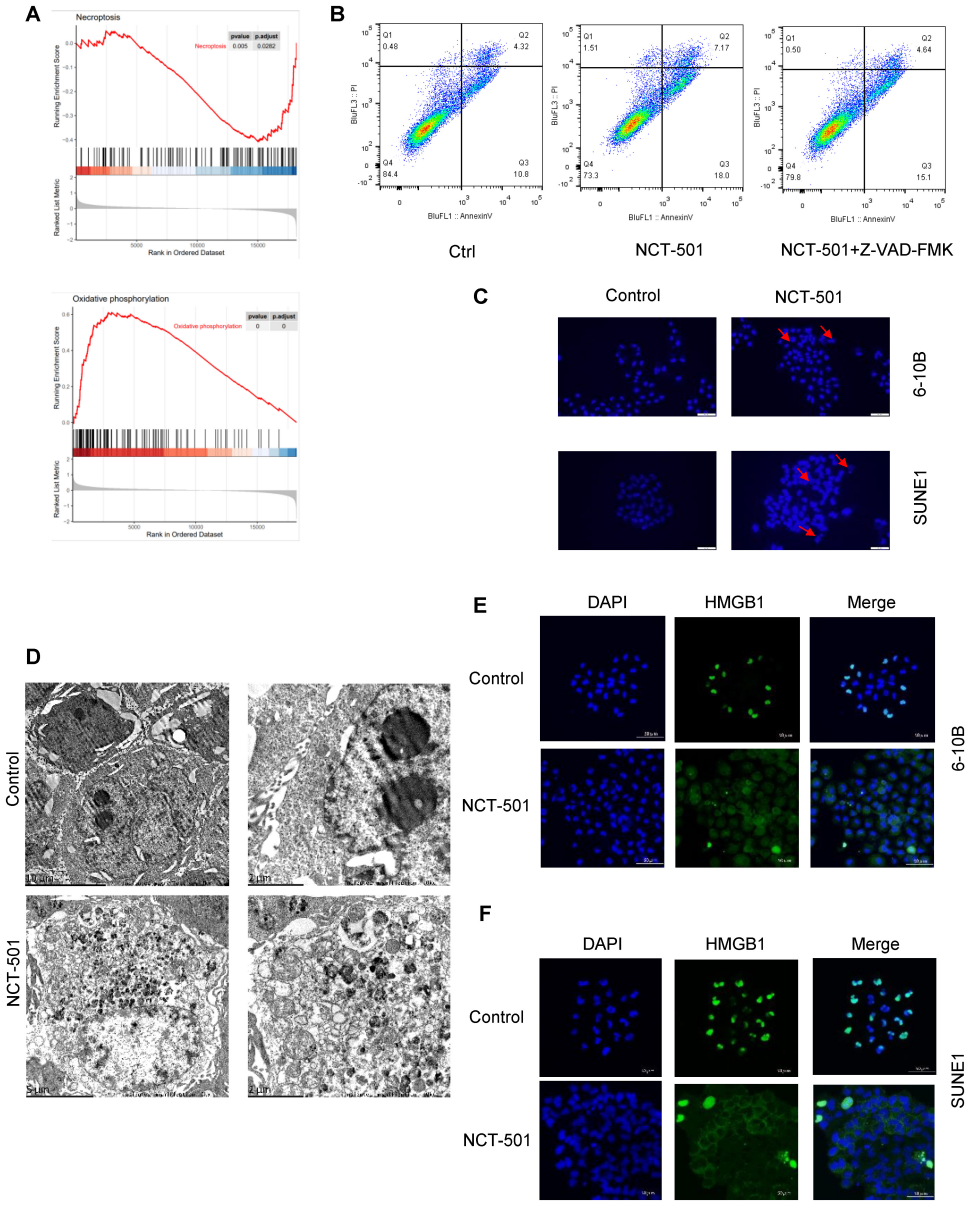
** NCT-501 targeting ALDH1A1 induced necroptosis in NPC. (A)** The GSEA enrichment analysis of GSE12452 dataset. **(B)** The flow cytometry analysis of cell death in SUNE1 cells pretreated with the pan-caspase inhibitor Z-VAD-FMK, and followed by exposing to ALDH1A1 inhibitor NCT-501. **(C)** Nuclear morphology evaluation of NCT-501-treated cells. **(D)** Transmission electron microscope observation of SUNE1 cells treated by NCT-501 and controls. **(E)** Immunofluorescence showed marked nuclear-to-cytoplasmic translocation of high mobility group 1 protein (HMGB1) under NCT-501 treatment.

In order to analyze the possible mechanisms involved in ALDH1A1 in NPC. We divided the GSE12452 dataset of NPC into ALDH1A^high^ and ALDH1A1^low^ groups according to the average level of ALDH1A1. And used GSEA enrichment analysis to analyze its main functions. We found that the expression level of ALDH1A1 was negatively correlated with tumor cell necroptosis and positively correlated with oxidative phosphorylation (Figures 4A). Next, we pretreated NPC cells with the pan-caspase inhibitor Z-VAD-FMK, and then they were exposed to ALDH1A1 inhibitor NCT-501. We discovered that the cells were not completely protected from 673A-induced cell death (Figures 4B). These data suggested that NCT-501-induced cell death was not entirely apoptosis-dependent, but partially non-apoptotic. Evaluation of NCT-501-treated cells stained with DAPI showed that nuclear volume increasing and fragmentation after NCT-501 treatment were consistent with necroptosis (Figure 4C). Likewise, transmission electron microscopy (TEM) revealed many morphological features of necrotic cells: swelling of cells, organelles, increased cell volume, enlarged mitochondria, rupture of the plasma membrane and release of cellular contents (Figure 4D), which Suggested that this was programmed cell necrosis or necroptosis. Immunofluorescence showed marked nuclear-to-cytoplasmic translocation of high mobility group 1 protein (HMGB1) under NCT-501 treatment (Figure 4E-F) 21.

## Discussion

Highly conserved aldehyde dehydrogenase (ALDH) family exerts cytoprotective effects in various tissues through its detoxification function. Besides, these enzymes catalyze the oxidation of retinol to retinal, a limiting step in retinoic acid synthesis that activates important cellular differentiation pathways 22. ALDH1A1 is one of the highly conserved isoenzymes of the aldehyde dehydrogenase family. In this study, we found that ALDH1A1 mainly played a carcinogenic role in NPC. Although the expression level of ALDH1A1 was down-regulated in NPC than normal tissues, its expression level was higher in patients with higher clinical stages than lower one. It was also associated with lymph node metastasis and poor survival status of patients. Multivariate regression analysis confirmed that it might be an independent indicator of poor prognosis in patients with NPC. This may be related to the function of ALDH1A1 itself. ALDH1A1, a member of the human aldehyde dehydrogenase family, metabolizes endogenously and exogenously produced aldehydes to their corresponding carboxylic acid derivatives to alleviate aldehyde stress. When ALDH1A1 is lowly expressed, it will cause the accumulation of aldehydes in the human body 1, 16. Aldehydes in sufficient quantities can cause cytotoxicity and carcinogenic effects 23. This theory is supported by several studies. Aldehyde dehydrogenase (ALDH) is a group of cytoplasmic enzymes that use nicotinamide adenine dinucleotide (NAD) as a coenzyme to oxidize aldehydes to corresponding carboxylic acids 3. Okudela et al. found that the expression level of ALDH1A1 in patients with non-small cell lung cancer was negatively correlated with carcinogenesis 24. In adenocarcinoma, the downregulation of ALDH1A1 is often more significant in high-grade, poorly differentiated tumors and tumors with stronger proliferative activity 24. But it was worth noting that the expression levels of ALDH1A1 in the higher clinical stages were higher than that in the lower clinical stages. And ALDH1A1 was related to the poor survival prognosis of patients.

Previous reports have linked ALDH, especially ALDH1A1, to stem cells, both in normal tissues, such as the hematopoietic environment, and in malignant tumors 25. In recent years, emerging efforts have focused on developing new therapies for CSC. Various strategies have been employed, including blocking pathways selectively activated in stem cells or agents targeting specific markers expressed on the surface of CSCs 26. Since high ALDH activity appears to be a hallmark of NPC CSCs 27. Our study found that the expression level of ALDH1A1 in NPC was correlated with the stem cell spheroid of NPC cells. In recent years, the emergence of ALDH1A1 inhibitors has attracted more attention. NCT-501 is a potent and selective theophylline-based inhibitor of aldehyde dehydrogenase 1A1 9. And ALDH1A1 inhibitor NCT-501 was able to inhibit stem cell spheroidize of NPC cells.

In addition, another key role exhibited by ALDH1A1 is the oxidation of retinal to retinoic acid (RA), forming a transcriptional regulator essential for cell growth and differentiation 28. The ALDH1A1 selective inhibitor NCT-501 can synergize with Olaparib to kill epithelial ovarian cancer cells carrying BRCA2 mutations 20. Small molecule inhibitor NCT-501 down-regulated ALDH1A1 expression and inhibited AKT-β-catenin signaling pathway, and the proliferation of esophageal squamous cell carcinoma cells 29. Similar results were provided in this study using the small molecule inhibitor NCT-501, which specifically inhibited ALDH1A1. In NPC, we used various concentrations (0-80 nM) of NCT-501, which were found to inhibit cell viability and the colony formation.

So, we further searched for its possible mechanism of inhibiting the proliferation of NPC cells. Firstly, the main possible role of ALDH1A1 in NPC was analyzed by taking GSE12452 as an example, and it was found that when ALDH1A1 highly expressed in tumors, GSEA enrichment analysis was negatively enriched in oxidative phosphorylation and positively enriched in necroptosis. At the same time, the ALDH1A1 inhibitor NCT-501 induced cell death did not all dependent on caspase in NPC cells. Further observation of nuclear morphology confirmed that this cell death was programmed necrosis or necroptosis, which was consistent with previous reports. ALDH1A inhibitor mediates the induction of necroptosis in ovarian cancer stem-like cells in part by inducing mitochondrial uncoupling proteins and reducing oxidative phosphorylation 16. Necroptosis is partly dependent on the induction of mitochondrial uncoupling protein (UCP) and is associated with inhibition of oxidative phosphorylation (OXPHOS) in CSCs 16. Necroptosis has been reported to be synergistic with chemotherapy and to be associated with reduced tumor initiating capacity and tumor eradication *in vivo* 30.

In this study, we found that although ALDH1A1 was down-regulated in NPC compared with non-cancerous tissues, it was highly expressed in high-stages patients. This suggested that the expression levels of ALDH1A1 at different degrees of tumor development changed dynamically. The ALDH1A1 inhibitor NCT-501 could not only inhibit the stem cell spheroid of NPC cells, but also suppress the proliferation of NPC cells via inducing necroptosis. Together, these data strongly support ALDH1A1 is a potential therapeutic target for NPC.

## Material and Methods

### Patients and samples

The Cancer Genome Atlas (TCGA) - The Genotype-Tissue Expression (GTEx) pan-cancer data were downloaded from the University of California Santa Cruz Xena Browser. Analysis of the whole genome data of pan-cancer cases by TCGA. Analysis of ALDH1A1 CNV frequencies of 27 cancer types by TCGA. Prognostic values of ALDH1A1 protein expression in 10 types of cancer patients were obtained from The Human Protein Atlas online database. Gene Expression Omnibus (GEO) Nasopharyngeal carcinoma samples were downloaded for the analysis about the ALDH1A1 expression.

### Ethical statement

All protocols were approved by The Second Xiangya Hospital of Central South University Ethics Review Board (Scientific and Research Ethics Committee, No. Y202/2014) and all research was performed in accordance with relevant guidelines/regulations. All research samples were obtained with written informed consent. If the patient is juvenile, a written consent will be signed by caretakers, or guardian on behalf of the juvenile participating in this study.

### Patient cohorts

Between 2011 and 2017, we collected 424 NPC patients, which diagnosed with NPC from The Second Xiangya Hospital, Central South University's. All tumors were assessed by expert pathologists using the WHO Nasopharyngeal carcinoma histological classification. The staging classification of the current analysis was carried out based on the criteria of the 8^th^ edition of the AJCC/UICC TNM staging system of NPC. The time from diagnosis to death, or the final known moment of survival, was used to calculate overall survival time. The Ethics Committee of Central South University's Second Xiangya Hospital accepted this study (No. Y202/2014), and all patients with written informed consent had access to comprehensive clinical and follow-up data.

### Exclusion and inclusion criteria

Eligible patients included in this article for are in accordance with the following inclusion criteria: (1) pathological confirmation of the diagnosis; (2) no residual cancer cells on microscopic examination; (3) none of them had received therapy targeting EGFR, PD1/PDL1 throughout the follow-up period; (4) at the time of the original procedure, no patients had received radiation or chemotherapy; (5) Complete follow-up data and clinicopathological data. The detailed clinic parameters of enrolled patients were presented in Table. Exclusion criteria included the following: (1) other treatments were used after the operation; (2) missing follow-up and deaths unrelated to tumor progression.

### Immunohistochemistry and scores

The immunohistochemistry experiment was conducted following the protocol our former study 31, 32. The dilution of primary antibody to ALDH1A1 was 1:300 (Mouse Monoclonal antibody, Catalogue 60171-1-Ig; Proteintech Group, Chicago, USA). Positive control slides were included in every experiment. The specificity of the antibody was determined with matched IgG isotype antibody as negative control. FSQ and WWY, who were blinded to the clinicopathological data, independently evaluated the expression of ALDH1A1 under a 200 × magnification light microscope. Staining intensity for ALDH1A1 was defined as: 0 (negative), 1 (weak), 2 (moderate), and 3 (strong). Staining extent was scored as 0 (no staining), 1 (1-25%), 2 (26-50%), 3 (51-75%), and 4 (76-100%), which depended on the percentage of stained cells. The staining intensity and extent were both based on tumor cells rather than lymphocytes. Overall score = intensity score × extent score. The total score was 0, 1, 2, 3, 4, 6, 8, 9 or 12. Referred to published literatures and combined with our practical situation, we made optimal cut-off levels as follows: and the score of 0 to 6 was defined as low expression, therefore 7 to 12 were considered high expression. Agreement between the 2 evaluators was 95%, and all scoring discrepancies were resolved through discussion 33, 34.

### Cell lines and cell culture

Cell lines (5-8F, 6-10B and HK1) used in this study were gifted by the Cancer Research Institute, Central South University. SUNE1 used in this study were gifted by Integrated Hospital of Traditional Chinese Medicine, Southern Medical University. All cell lines were recently authenticated using short tandem repeat (STR) profiling by Microread Gene Technology (Beijing, China). The human NPC cell lines (5-8F, 6-10B and SUNE1) were cultured in DMEM medium (Biological Industries, Israel) supplemented with 10% fetal bovine serum (Gibco, USA), and HK1 were cultured in RPMI-1640 medium (Biological Industries, Israel). The culture dishes were placed in the incubator at 37 °C, 5% CO_2_.

### CCK8 assay and colony formation

Cell survival rates were estimated by the CCK-8 assay (Bimake, USA). 3000 cells were counted and seeded in 96-well plates with 100 μl medium each well. CCK-8 reagents (Bimake, USA) were added at every at 24h for five days. Theoptical density was estimated at 450 nm wavelength by Multilabel Plate Reader (SpectraMax iD3, Molecular Devices). For colony formation assay, 3000 cells were counted and seeded in 24-well plates, cells were fixed and stained with 0.1% crystal violet staining solution to visualize colonies after about 14 days.

### Stem Cell Sphere Formation Assay

Cells (1×10^4^ cells/well) were plated in 6-well plates with ultra-low adherence (Corning, USA) and cultured in DMEM/F12 medium, supplemented with B27, 20 ng/ml EGF and 20 ng/ml bFGF for 14 days to form spheres 29.

### Western blot analysis

Western blotting was performed according to the standard protocol 35 with the following antibodies: ALDH1A1 (Proteintech Group).

### Transmission electron microscopy

The cells cultured in 6-well plates were digested and collected. And the cells were fixed in PBS solution containing 2.5% glutaraldehyde for 24 h. After being washed in 0.1 M PBS, the cells were treated with 0.1% Millipore-filtered cacodylate-buffered tannic acid, postfixed with 1% buffered osmium, and stained with 1% Millipore-filtered uranyl acetate. After dehydration and embedding, samples were incubated in a 60 °C oven for 24 h. Digital images were obtained using a transmission electron microscope.

### Statistical methods

Statistical analyses were performed by log-rank test, Chi-square test, multivariate Cox regression analysis, and student's t-test as appropriate using SPSS for Windows (18.0; SPSS, Inc.) and GraphPad Prism (Prism 8.0; GraphPad Software Inc.) packages. A *P* value < 0.05 was considered significant. Error bars indicate the standard deviation in all the Figures. *P < 0.05, **P < 0.01, ***P < 0.001 by two-tailed t-test.

## Figures and Tables

**Table 1:** Analysis of the association between expression ALDH1A and clinicopathological features of NPC (n=424)

Clinicopathological features (n=424)	ALDH1A1		
	Low (%)	High (%)	*P*-value
**Age (years)**			
≤55 (n=320)	199 (62.2)	121(37.8)	0.250
> 55 (n=104)	58 (55.8)	46 (44.2)	
**Gender**			
Male (n=309)	190 (61.5)	119 (38.5)	0.577
Female (n=115)	67 (58.3)	48 (41.7)	
**Tumor stages**			
Stage 1-2 (n=220)	132 (60.0)	88 (40.0)	0.847
Stage 3-4 (n=204)	125 (61.3)	79 (38.7)	
**LN status**			
No LNM (n=38)	29 (76.3)	9 (23.7)	0.038*
LNM (n=386)	228 (59.1)	158 (40.9)	
**Metastasis**			
No (n=370)	225 (60.8)	145 (39.2)	0.882
Yes (n=54)	32 (59.3)	22 (40.7)	
**Clinical Stages**			
I-II (n=54)	44 (81.5)	10(18.5)	0.001*
III-IV (n=370)	213 (57.6)	157 (42.4)	
**Survival status**			
Alive (n=314)	206(65.6)	108 (34.4)	<0.001*
Dead (n=110)	51 (46.4)	59 (53.6)	

*Chi-square test, statistically significant difference (*P < 0.05, **P < 0.01). Abbreviations: H, High expression; L, Low expression; LNM, lymph node metastasis.

**Table 2:** Summary of multivariate of Cox proportional regression for overall survival in 424 cases of NPC

Parameter	B	SE	Wald	Sig.	Exp (B)	95.0% CI for Exp (B)	
						Lower	Upper
Age	.398	.210	3.587	.058	1.488	.986	2.246
Gender	-.119	.221	.288	.592	.888	.575	1.370
Tumor stages	.298	.196	2.322	.128	1.347	.918	1.977
LN status	1.226	1.029	1.418	.234	3.406	.463	25.612
Metastasis	.773	.228	11.456	.001**	2.167	1.385	3.391
Clinical Stages	2.554	1.007	6.429	.011*	12.856	1.785	92.564
ALDH1A1	.449	.192	5.486	.019*	1.567	1.076	2.282

Abbreviations: CI, confidence interval; LNM, lymph node metastasis; NPC: nasopharyngeal carcinoma. Note: multivariate analysis of Cox regression, *P < 0.05, **P < 0.01, ***P < 0.001.
